# Account of the Intermittent Fever, as It Prevailed among a Native Gang at the Island of Erromanga, One of the New Hebrides Group

**Published:** 1832-03

**Authors:** George Bennett

**Affiliations:** Member of the Royal College of Surgeons in London, &c.


THE LONDON

### Medical and Physical Journal.

397, VOL. LXVII.] MARCH 1832. [69, *New Series.*

"For many fortunate discoveries in medicine, and for the detection of numerous errors, the world is indebted to the rapid circulation of Monthly Journals; and there never existed any work to which the Faculty, in Europe and America, were under deeper obligations than to the 'Medical and Physical Journal of London,' now forming a long but invaluable series."—RUSH.

### ORIGINAL PAPERS, AND CASES

### OBTAINED FROM PUBLIC INSTITUTIONS AND OTHER AUTHENTIC SOURCES.

### INTERMITTENT FEVER.

*Account of the Intermittent Fever, as it prevailed among a Native Gang at the Island of Erromanga, one of the New Hebrides Group.* By GEORGE BENNETT, Esq., Member of the Royal College of Surgeons in London, &c.

INTERMITTENT fever prevailed to an extensive degree among a gang composed of natives of Tongatabu and Rótuma, who had been landed (for the purpose of cutting sandal wood) on the island of Erromanga, one of the New Hebrides group. The first encampment was formed at Marekini Bay, which was backed by high hills, covered with wood. During the stay, and just previous to the arrival of the ship Sophia, (of which ship I had medical charge,) a few cases occurred, which were readily cured by the sulphate of quinine: the exciting cause of this fever I then attributed, in some degree, to the natives, when returning from the mountains in a state of profuse perspiration, bathing immediately in the river. On the departure of the ship, (September 3, 1829,) no fresh cases had occurred, and the schooner Snapper remaining with the gang at the island, to procure supplies of provision, &c., some sulphate of quinine was left with the commander, with directions how to administer it to any fresh cases that might occur.

It was not, however, until our return, on the 6th of March, 1830, to Gulantap Bay, on the same island, (to which place the gang had removed only a short time before our arrival, having previously been from Marekini to Cook's Bay,) that we found to what a fatal extent the sickness had prevailed: they informed us that, soon after our departure, in September

1829, the rainy season set in,* but that the fever did not prevail so much at the time of the rains as subsequently. I found, on landing, that the gang had encamped themselves, formed a stockade, and erected their habitations in a dense jungle, near the beach, it was true, but sheltered from the prevailing east-south-east tradewind by the trees, and in the jungle large trees and rank herbage abounded: the huts were built close together, without any regard to ventilation; and they permitted the remains of their vegetable food to rot about their habitations, occasioning a disagreeable and unhealthy effluvia. At night the landbreeze usually set in, blowing over an extent of jungly country; and close above them were lofty hills, covered with a dense vegetation.

Although this fever prevailed to its greatest and most fatal extent after the rains, they cannot be considered the sole exciting cause of this fever, as it occurred in several cases previous to the rains, and some Tahitans were also subsequently attacked by it at the island of Annatom, some time after the fine-weather season had commenced. At Marekini Bay, the prevailing winds were usually over the land.

Besides the causes just mentioned, the natives slept on the floor of their huts, which was merely covered by plaited sections of the cocoa-nut frond, which rendered them more liable to inhale the noxious exhalations arising from the decomposition of so much vegetable matter. I observed no marshy land in the vicinity of the stockade; the soil was rich, consisting of decayed vegetable matter, in which a luxuriant vegetation grew, watered by a few small rills, which trickled down from the base of the lofty mountains towards the sea; and most excellent fresh water could be procured by digging in the sand on the beach, above highwater mark. The portions of land cultivated by the aborigines were surrounded by a density of vegetation, so characteristic of a tropical country; only that portion being cleared which was immediately required for cultivation. When I entered the jungle in the vicinity of the stockade, on a botanical excursion, I experienced such an oppressive heat and powerful smell from the dense herbage as to occasion my return.

The natives of Rótuma forming the gang were landed on the island by the schooner Dhaule, after the rainy season; but the sickness and deaths among them were greater than among the natives of Tongatabu, who had been for a much

* Captain Hardy, of the schooner Snapper informed me that the rainy season commenced in September, and terminated about the middle of December: it was after the rainy season that the fever prevailed to the greatest extent.

longer time on the island. The causes of the greater mortality among the Rótuma natives, in comparison with those of Tongatabu, I attribute in some degree to the weaker constitutions of the former, compared with the latter; the natives of Tongatabu being muscular, powerful men, capable of enduring any fatigue; whilst the natives of Rótuma were weak, and unable to endure the fatigue attendant on ascending hills to cut the sandal wood, and bring down a load; and the Rótuma natives had so lost the vital energy, through the disease, as to be beyond the influence of medicine to restore them.

The probable causes which produce intermittent fever have not yet been accounted for: the miasmata arising from marshes has been considered one of the causes. Although we find it produced in the vicinity of marshes, we also find it occur where no marshes exist. Dr. Ranken observes, "There are no swamps, nor indeed perceptible water, in the most deadly tracts of the Nepaul and Malwa forests, though their unventilated dells teem with effluvia of the greatest virulence. The medical topography of several European locations also exemplifies the fact, though less forcibly; but I have never traced miasm in its effects from sand, which forms the soil, almost entirely, of our healthiest stations."* One of the attributable causes might be the unventilated space in which the native habitations had been constructed, and in which they slept, surrounded by herbage and decaying and decayed vegetable matter, aided by occasional ill supplies of provisions, when they were retarded by the hostility of the natives. Dr. Rankin, however, in my opinion, proceeds too far, when he considers that trees, or any vegetables, growing in towns or villages, are injurious to human life by their tendency to promote several diseases, especially fevers; and by proposing that gardens, plantations, &c. should be formed distant from cities, towns, &c.: it is in a dense vegetation, where the sun hardly ever penetrates, destitute of ventilation, that the constant falling of the leaves, decomposing under a tropical sun, generates a deadly miasmata; for we do not find it existing in cultivated districts, the trees being planted in such a manner that the sun can exert his influence, and every part is well ventilated: for, as Dr. Arnott observes, "in the vegetable creation, the beautiful green leaf or delicate flower is merely a broad and tender expansion of surface for the contact of the vivifying air. Animals give out to the atmosphere a substance which

* Med. and Phys. Trans. of Calcutta, vol. iii. p. 303.

vegetables absorb, and vegetables, by removing this, fit the air again for the use of animals; so that, upon the whole, in the various changes of nature, there is, in the atmosphere, a perfect balancing of absorption and exhalation, preserving the mass in an uniform state, constantly fit for its admirable purposes;" but Mr. STEVENSON observes, that, "in the growth and productions of plants, unnatural and diseased actions take place, from the unseasonable or superabundant supply of water, the absence of light, the want of proper digestion and assimilation in their juices, and the proper combination of their elementary principles, carbon, hydrogen, and oxygen. And if it be admitted that, in the growth of vegetables, oxygen and carbonic acid gas are alternately evolved under certain circumstances, may we not infer, that in low, marshy, and jungly countries, during the period of the rains, and while the atmosphere is thick, dull, and obscure, vegetation, without the stimulus of light, will be attended with a great evolution of fixed air, which must tend, in a proportionate degree, to reduce the purity of the atmosphere? or may we not attribute to a dense and crowded vegetation, in dark and gloomy situations, deleterious changes in our atmosphere, similar to those which arise from the crowded state of animals in dark and ill-ventilated dungeons? both emit a gas deleterious to life, and the growth of each is checked, if not destroyed, in such circumstances."*

On the 21st and 22d of January, 1830, the schooner Dhaule, Captain Bancroft, landed 163 natives from the island of Rótuma at this island, and, on our arrival on the 6th of March, eighty were already dead; three having been killed by the natives, and the remainder dead from fever. The Sandwich islanders at Wiriau, or Cook's Bay, were suffering also from fever, and dying in numbers. Several of the Rótuma natives died suddenly; but, as these cases occurred previous to my arrival, I had no opportunity of seeing them. It seems the native would previously appear in good health, be seized suddenly with shivering, giddiness, and in a short time would be found dead; proceeding, no doubt, from effusion on the brain, consequent on the first attack of intermittent fever.†

The deaths among the natives of Tongatabu were but few, the principal mortality being among those of Rótuma. Nu-

* Med. and Phys. Trans. of Calcutta, vol. iii. p. 100—1.

† It has been stated in our medical works, that "such a depression of strength has been known to take place on the attack of an intermittent, as to cut off the patient at once, but an occurrence of this kind is very uncommon." Can the sudden deaths of the Rótuma natives be attributable to this cause?

merous cases which came under my charge had been suffering three months from paroxysms of intermittent fever. Having fortunately a large supply of the sulphate of quinine on board, I administered doses of from seven to twelve grains, and its success was complete, excepting in those cases in which the debility was excessive; for, although it removed the paroxysm, it had no effect on the visceral disease, which terminated the existence of the patient who had been long suffering from paroxysms of fever.

From the sickness that prevailed, the invalids were speedily removed on board the ship, where, the between-decks being clear, they were well accommodated; and on the 9th of March we had removed the whole of the sick on board, and sailed for Wiriau, or Cook's Bay, to observe the state of the Sandwich islanders.

The number of sick on board was as follows: 4 Fidgeans, 9 New Zealanders (including two females), 2 Tahitans, 2 Europeans, 101 natives of Tongatabu, 60 natives of Rótuma; making a total of 193. Besides these, we had 200 natives of Rótuma in health on board, who had been brought to Erromanga, being ignorant at the time of the sickness that prevailed.

The original number landed on the island was as follows: 9 New Zealanders (including two females), 2 Tahitans, 2 Europeans, 6 Fidgians, 163 natives of Rótuma, 107 Tonga natives; total, 288. Of these, among the Tongatabuans, Tai was killed by an arrow through the heart; Lau killed by the natives.

On the 8th, one of the Rótuma natives dying on board, I wished to inspect the body, but first thought it better to ask permission of one of the chiefs on board, by addressing him in his own figurative style of speech, "that my heart bled to see his people die; that I wished to see the cause, and look inside the man, by which I might be enabled to cure others:" he gave his consent, considering "that doctor know every thing, and Rótuma chief know nothing," &c. Externally the body had a very emaciated appearance, and the tunica conjunctiva was tinged with bile; internally, the thoracic viscera were healthy; the liver was somewhat enlarged, and rather paler than usual, but of firm texture; the gall-bladder was filled with dark-coloured viscid bile; the spleen was much enlarged, soft, breaking readily under the fingers, having the cells filled with dark, grumous blood; the lower intestines exhibited several small ulcerative patches on the internal coats. There was some curiosity shewn among the natives during the inspection of the body, and several of them were present, but there was no manifestation of dislike, or any particular remark made on the subject.

When the aborigines were on friendly terms with the gang, and saw some of them suffering from the paroxysms of fever, they expressed much surprise; from which I should be inclined to consider the fever as unknown to them.

We found the Sandwich islanders suffering severely, and the deaths among them were very numerous; and, from a letter I received recently from the Sandwich islands, I was informed that, out of the whole gang that sailed in the Becket schooner, there returned only Kaupéné (the widow of the chief Manuia,) and a boy.

During the sickness at Erromanga, the New Zealanders having observed that, among the natives of Tongatabu, there were but few deaths, whilst, among those of Rótuma, the mortality was great, attributed it to the superior power of the Tongatabuan's "new God," as they were Christians; and they consequently, most piously, joined the Tonga natives in their prayers; but when they came on board, and had recovered from their illness, they did not consider it requisite to trouble themselves by praying any more, so they left the Tonga natives to sing hymns and repeat prayers by themselves.

On the 11th of March we left Cook's Bay for the island of Rótuma, to land the natives we had brought from thence, those who were sick, and also the Tongatabu natives, previous to their being removed to their own island. During the passage, we were much retarded by light winds and calms; the between-decks were frequently cleaned and fumigated, every attention being directed to cleanliness on board. The deaths were numerous daily, but from cases before considered as hopeless, all of which terminated in colliquative diarrhœa. Of the sixty natives of Rotuma brought on board, only twenty-nine had paroxysms; the others were suffering, and subsequently died, from debility, attended by colliquative diarrhœa: most of the cases that came under my charge had been ill from two to six weeks, and some even for three months; but, wherever the debility was not too great, the sulphate of quinine was given with great advantage; but in hot climates it is found that the debilitating effects of the disease are very great, and reattacks very liable to occur.

On the 20th of March we arrived at the island of Rótuma, and landed the sick and convalescents: only twenty-three of the Rótuma natives had survived. Among the natives of Tongatabu the mortality was not so great on board, but the unfavorable state of the weather at that island, and their carelessness in exposing themselves to the weather, brought on reattacks among those who had been cured, and destroyed many of those who were suffering from paroxysms. The number among the natives of Tongatabu that died on board and previous to our departure from Rótuma, were thirty-two, most of whom died on shore, where I had seen them exposing themselves constantly to the rain.

In pressing the splenic region of the unfavorable cases, they shrank, and complained of pain, but there was no external appearance of enlargement of that organ. Headach was produced by the quinine in a few cases among those of plethoric habits, but was speedily removed by the administration of purgatives.

CASES.

1. Mousmong, a young man, native of the island of Rótuma, had been suffering from quotidian paroxysms of intermittent fever, about a fortnight previous to his coming under my charge, on the 6th of March, 1830. On the 7th, the paroxysms came on in the afternoon, at the commencement of which he took six grains of quinine, and the same dose was repeated during the paroxysm: it shortened (according to his statement) the usual duration of the paroxysm. The following day, a purgative was administered: a slight paroxysm was felt, for which five grains were given.

9th. No paroxysm: a bark mixture ordered, wine and nourishing diet; and on the 16th he was discharged cured.

2. Richard Richards, aged thirty, of New South Wales. He was a young man of spare habit, and came under my care on the 7th of March, 1830. He came in the ship Sophia from New Zealand, and was left, at his own request, to superintend the gang at the island of Erromanga, on the 2d of September, 1829: when he came on board on the 7th, he was in a very emaciated state, having been for two months suffering from quotidian paroxysms of fever: his legs were swollen, as also the testicles, and the debility was excessive. The paroxysms came on with great severity, leaving at their termination a derangement of intellect for a short period.

March 8th. About noon the paroxysms came on, (being the usual hour at which they appeared:) eight grains of the quinine were given at the commencement, and five grains during the paroxysm. His bowels were freely open. Nourishing diet, and wine ordered daily.

11th. He has had no paroxysm since the 8th: a quinine mixture ordered. The tumefaction of the legs and testicles much reduced, and he was able to go on deck for a short time every day.

On the 12th, no paroxysm, but has a troublesome diarrhœa, for which a chalk mixture was ordered.

14th. Diarrhœa still continues, with great debility: the chalk mixture ordered to be repeated, with increased doses of kino and opium.

17th. He has been slightly relieved by the astringent mixtures, but the debility is very great. Has had no return of the paroxysms.

19th. The diarrhœa is checked for a short period, but soon returns; pulse weak, and breathing very hurried. He still has nourishing diet and wine.

21st. An opium draught succeeds in checking the diarrhœa for a short time. The debility and emaciation is very great; the tongue is covered by brown incrustations; and he appears gradually sinking, from the debility consequent on the colliquative diarrhœa.

29th. Extreme debility; discharges his fæces involuntarily, and appears rapidly sinking.

On the 30th of March, the ship was driven on shore at the island of Rótuma: he was kept on board with us during the night, when he became insensible, and expired about midnight. On the following morning the weather moderated, and he was taken on shore, and buried.

*London; February*, 1832.

